# Comparison of Emergency Medical Dispatch Systems for Performance of Telecommunicator-Assisted Cardiopulmonary Resuscitation Among 9-1-1 Callers With Limited English Proficiency

**DOI:** 10.1001/jamanetworkopen.2021.6827

**Published:** 2021-06-02

**Authors:** Stephen Sanko, Siyu Feng, Christianne Lane, Marc Eckstein

**Affiliations:** 1 Division of Emergency Medical Services, Department of Emergency Medicine, Keck School of Medicine of the University of Southern California, Los Angeles; 2 Emergency Medical Services Bureau, Los Angeles Fire Department, Los Angeles, California; 3 Division of Biostatistics, Department of Preventative Medicine, Keck School of Medicine of the University of Southern California, Los Angeles

## Abstract

**Importance:**

Increasing bystander cardiopulmonary resuscitation (CPR) among racial/ethnic minority groups and culturally underserved populations is a key strategy in improving health care disparities in out-of-hospital cardiac arrest.

**Objective:**

To ascertain whether implementation of the Los Angeles Tiered Dispatch System (LA-TDS) was associated with improved performance of telecommunicator-assisted CPR (T-CPR) among 9-1-1 callers with limited English proficiency in the City of Los Angeles.

**Design, Setting, and Participants:**

This cohort study compared emergency medical services–treated, nontraumatic out-of-hospital cardiac arrest calls using the Medical Priority Dispatch System (MPDS) from January 1 to March 31, 2014, with calls using LA-TDS from January 1 to March 31, 2015. Trained data abstractors evaluated all 9-1-1 audio recordings for the initiation of T-CPR and the elapsed time to predefined events. Data were analyzed between January and December 2017.

**Main Outcomes and Measures:**

The primary outcome was the prevalence of T-CPR among 9-1-1 callers with limited English proficiency for field-confirmed nontraumatic cardiac arrests. Additional outcomes included T-CPR among callers with English proficiency and the elapsed time until key events in the call.

**Results:**

Of the 1027 emergency medical services calls during the study periods, 597 met the inclusion criteria. A total of 289 calls (48%) were made using MPDS (263 callers with English proficiency, and 26 callers with limited English proficiency), and 308 calls (52%) were made using LA-TDS (273 callers with English proficiency, and 35 callers with limited English proficiency). No differences between MPDS and LA-TDS cohorts were found in age, sex, known comorbidities, arrest location (private vs public), or witnessed status. The prevalence of T-CPR among callers with limited English proficiency was significantly greater using LA-TDS (69%) vs MPDS (28%) (odds ratio [OR], 5.66; 95% CI, 1.79-17.85; *P* = .003). For callers with English proficiency, the prevalence of T-CPR improved from 55% using MPDS to 67% using LA-TDS (OR, 1.66; 95% CI, 1.15-2.41; *P* = .007). With LA-TDS, callers with limited English proficiency had a significant decrease in time to recognition of cardiac arrest (OR, 0.59; 95% CI, 0.41-0.84; *P* = .005) and dispatch of resources (OR, 0.71; 95% CI, 0.54-0.94; *P* = .02).

**Conclusions and Relevance:**

The LA-TDS compared with MPDS was associated with increased performance of T-CPR for out-of-hospital cardiac arrests involving 9-1-1 callers with limited English proficiency. Further studies are needed in communities with a predominance of people with limited English proficiency to characterize bystander response, promote activation of the chain of survival, and clarify the precise elements of LA-TDS that can improve T-CPR performance.

## Key Points

**Question:**

Did implementation of a new dispatch system change telecommunicator-assisted cardiopulmonary resuscitation (T-CPR) for patients in cardiac arrest involving 9-1-1 callers with limited English proficiency?

**Findings:**

In this cohort study of 597 emergency calls for cardiac arrest, compared with the Medical Priority Dispatch System that was used for 25 years in the City of Los Angeles, the new Los Angeles Tiered Dispatch System was associated with an immediate increase in the prevalence of T-CPR and decreased times to cardiac arrest recognition and first chest compression when callers had limited English proficiency.

**Meaning:**

This study found an association between the Los Angeles Tiered Dispatch System and improved emergency care in cardiac arrest cases involving 9-1-1 callers with limited English proficiency; further studies are needed in this group to increase activation of the chain of survival and improve health outcomes in cardiac arrest.

## Teaser

This cohort study evaluates the prevalence of telecommunicator-assisted cardiopulmonary resuscitation among 9-1-1 callers with limited English language skills using 2 emergency medical services dispatch systems.

## Introduction

Early provision of cardiopulmonary resuscitation (CPR) is a key factor in survival from out-of-hospital cardiac arrest (OHCA).^1,2,3^ Although the benefit of bystander CPR has been well established, previous literature indicates that less than 30% of patients in Los Angeles (California) who experienced cardiac arrest received bystander CPR,^4^ including only 13% of cases involving African American or Latino patients.^5^ Telecommunicator-assisted CPR (T-CPR), in which 9-1-1 call takers quickly identify cases of possible cardiac arrest and provide CPR instructions, has been associated with a substantially increased rate of bystander CPR^6,7^ and improved survival.^8,9,10^

The Los Angeles Fire Department (LAFD), the second largest municipal 9-1-1 emergency medical services (EMS) agency in the United States, used the Medical Priority Dispatch System (MPDS) for more than 25 years (1989-2014). In late 2014, the LAFD developed a new series of scripted questions that decreased the number of questions needed to identify individuals experiencing a potential cardiac arrest and lowered the threshold for providing T-CPR. This new program, called the Los Angeles Tiered Dispatch System (LA-TDS), went into use on December 1, 2014. Since then, LA-TDS has substantially decreased call processing times for time-critical 9-1-1 emergencies,^11^ decreased undertriage in cases of field-confirmed OHCA,^12^ and improved the global rates of T-CPR.^13^

The objective of this cohort study was to ascertain whether implementation of LA-TDS was associated with increased prevalence of T-CPR among 9-1-1 callers with limited English proficiency in the City of Los Angeles. The predefined secondary hypothesis before LA-TDS implementation was that there would be no difference in the prevalence of T-CPR between MPDS and LA-TDS cohorts.

## Methods

This cohort study was a predefined secondary analysis of a before-and-after intervention study^14^ that compared telecommunicator management of OHCA at the City of Los Angeles 9-1-1 Dispatch Center during 2 separate 3-month periods: (1) between January 1 and March 31, 2014, using MPDS and (2) between January 1 and March 31, 2015, using LA-TDS. The present retrospective study was approved by the institutional review board of the University of Southern California, which granted a waiver of informed consent because the research involved no more than minimal risk to participants, the research could not be carried out practicably without the waiver, and the waiver would not adversely affect the rights and welfare of the participants. We followed the Strengthening the Reporting of Observational Studies in Epidemiology (STROBE) reporting guideline.^15^

The LAFD is the sole 9-1-1 EMS system for the entire City of Los Angeles. As an all-life hazard emergency response agency, the LAFD provides tiered basic and/or advanced life support response using a combination of ambulances and nontransporting fire engine or fire truck resources and performs all of its own transports to the hospital.

The LAFD 9-1-1 Dispatch Center is a secondary public safety answering point that receives approximately 1.1 million calls annually, resulting in more than 400 000 EMS incidents per year according to internal LAFD data. The dispatch center is staffed by sworn firefighters who have a minimum of basic life support training and 2 years of field EMS experience. Callers interact with a single telecommunicator (ie, a 9-1-1 call taker who is otherwise known as a dispatcher) who uses LA-TDS scripted and semiscripted questions to arrive at a dispatch code, which is then entered into a homegrown Los Angeles computer-assisted dispatch system. Each dispatch code is assigned an algorithm of resource assignments on the basis of location, level of service, and need for additional personnel on scene. Telecommunicators work 24-hour platoon duty shifts and field approximately 100 calls per shift. No change to the dispatch center, computer-assisted dispatch system, dispatch or ambulance staffing, dispatch or field time-stamping procedures, or electronic health record was observed during the study periods. Further information on telecommunicator training and accreditation has been previously published.^11,12,14^

Cases were selected by retrospectively reviewing LAFD electronic health records of patients who were diagnosed in the field with cardiac arrest, and filtering was based on Utstein elements^16^ as well as study inclusion and exclusion criteria. In this study, we included all LAFD-attended cardiac arrests with attempted resuscitation. Incidents were excluded if they were of obvious traumatic cause, occurred in a medical clinic or nursing home, were handled by non-LAFD dispatch centers, were witnessed by EMS personnel, or if CPR was in progress before the 9-1-1 call.

For all incidents that met the inclusion criteria, personnel who were blinded to the study hypothesis located the recorded 9-1-1 call for that incident. Data were provided to a non-LAFD biostatistician (C.L.) from the Southern California Clinical and Translational Science Institute who assigned a unique nonsequential case number to each audio recording. Four trained non-LAFD abstractors listened to all recorded calls and used strict criteria to ascertain whether T-CPR was initiated and to identify the elapsed time from the start of the call until key events in the call (eg, delivery of CPR instructions, delivery of first chest compression). Twenty calls from each comparison group were screened by all abstractors to assess interrater reliability for study elements (κ = 0.76).

The LAFD used MPDS from January 1, 1989, through November 30, 2014. For MPDS study period (January 1 to March 31, 2014), all emergency calls were answered by fully qualified and MPDS-accredited telecommunicators. From October 1 to November 30, 2014, while using MPDS on the dispatch floor, telecommunicators were trained on LA-TDS during their off time. The LA-TDS was implemented for all telecommunicators at all dispatch consoles on December 1, 2014, and was used exclusively during LA-TDS study period (January 1 to March 31, 2015).

The primary outcome of this study was the prevalence of T-CPR among 9-1-1 callers with limited English proficiency for field-confirmed nontraumatic cardiac arrests. This prevalence was defined as the number of patients who received telecommunicator-assisted chest compressions during a 9-1-1 call with callers with limited English proficiency divided by the total number of callers with limited English proficiency who met the inclusion criteria and did not meet 1 or more of the exclusion criteria. A caller was considered to have limited English proficiency if, in the best judgment of the 9-1-1 audio recording reviewer, the person did not speak English as a primary language and thus had limited ability to speak and understand English.^17^

Outcome definitions, abstractor approach to minimizing potential bias, data handling, and statistical methods have been described elsewhere.^14^ Race and ethnicity of patients were classified by the EMS personnel on scene.

### Statistical Analysis

Univariate characteristics of patients, electronic records of calls, and call review outcomes were described using mean (95% CI) for continuous outcomes and No. (%) for categorical outcomes. Time outcomes were examined for normality and nonparametric statistics and were investigated for any serious deviations from normality. Comparisons between LA-TDS and MPDS were made using *t* tests (for normally distributed continuous outcomes), Kruskal-Wallis test (for non-normally distributed continuous outcomes), or χ^2^ tests (for categorical outcomes). Timed events were compared using nonparametric tests that examined the median, the range, and the distribution of times. For the primary outcome of prevalence of telecommunicator-assisted chest compressions, α = .05. Ad hoc examination was performed using logistic regression to ascertain whether language proficiency moderated the improvements in T-CPR between MPDS and LA-TDS.

Analyses were performed with IBM SPSS Statistics for Macintosh, version 24.0 (IBM Corp). Data were analyzed between January and December 2017.

## Results

For the study periods of January 1 to March 31, 2014 (MPDS cohort), and January 1 to March 31, 2015 (LA-TDS cohort), a total of 1027 EMS-treated cardiac arrest cases occurred, of which 13 audio recordings could not be recovered and 417 cases were excluded per study protocol (Figure). A description of excluded cases in each cohort has been described.^14^ Of the 597 emergency calls that met the inclusion criteria, 289 (48%) were in MPDS cohort (263 callers with English proficiency, and 26 callers with limited English proficiency) and 308 (52%) were in LA-TDS cohort (273 callers with English proficiency, and 35 callers with limited English proficiency). Patient and event characteristics broken down by language proficiency and dispatch system cohorts are shown in Table 1.

**Figure.**
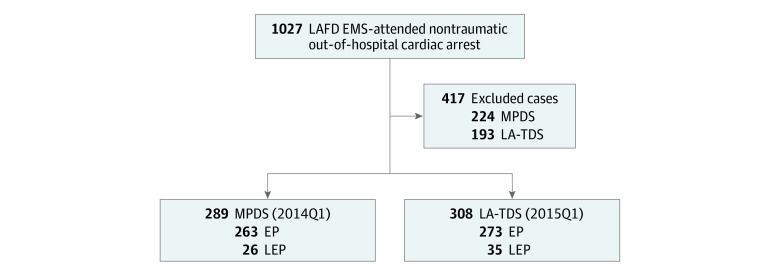
Flow Diagram of 9-1-1 Call Reviews of Out-of-Hospital Cardiac Arrests All 9-1-1 emergency medical services (EMS)–treated cardiac arrest cases from January 1 to March 31, 2014 (using the Medical Priority Dispatch System [MPDS]), and from January 1 to March 31, 2015 (using the Los Angeles Tiered Dispatch System [LA-TDS] cohort), were considered for study inclusion. Cases were excluded if they met 1 or multiple exclusion criteria.^14^ EP, callers with English proficiency; LAFD, Los Angeles Fire Department; LEP, callers with limited English proficiency; and Q1, quarter 1.

**Table 1.** Patient and Event Characteristics of the Medical Priority Dispatch System (MPDS) Cohort vs the Los Angeles Tiered Dispatch System (LA-TDS) Cohort

Variable	Callers with English proficiency, No. (%) (n = 536)			Callers with limited English proficiency, No. (%) (n = 61)			*P* value^a^
	MPDS cohort (n = 263)	LA-TDS cohort (n = 273)	*P* value^a^	MPDS cohort (n = 26)	LA-TDS cohort (n = 35)	*P* value^a^	
Male sex	166 (63)	174 (64)	.93	11 (44)	20 (57)	.32	.07
Female sex	96 (37)	99 (36)		14 (56)	15 (43)		
Age, median (IQR), y	65 (24)	65 (26)	.80	66 (18)	71 (25)	.58	.07
Age, y							
≥65	133 (51)	137 (50)	.93	13 (50)	21 (60)	.44	.43
<65	130 (49)	136 (50)		13 (50)	14 (40)		
Ethnicity							
Non-Hispanic	56 (55)	50 (65)	.20	1 (11)	2 (17)	.60^c^	<.001^c^
Hispanic	38 (37)	19 (25)		6 (67)	5 (42)		
Others^b^	8 (8)	8 (10)		2 (22)	5 (42)		
Race							
White	53 (52)	44 (57)	.76^c^	7 (78)	4 (33)	.16^c^	.004^c^
Black	41 (40)	25 (32)		0	3 (25)		
Pacific Islander	3 (3)	3 (4)		0	1 (8)		
Asian	5 (5)	5 (6)		2 (22)	4 (33)		
Unknown	161 (61)	196 (72)		17 (65)	23 (66)		
Weight, median (IQR), lb	170 (55)	170 (55)	.85	150 (125)	160 (60)	.67	.002
Disease history							
Asthma	20 (8)	15 (5)	.32	0	0	NA	.04^c^
Cancer	13 (5)	8 (3)	.23	0	2 (6)	.50^c^	>.99^c^
Cardiac	82 (31)	78 (29)	.51	2 (8)	10 (29)	.05^c^	.10
Chronic respiration failure or emphysema	14 (5)	13 (5)	.77	0	2 (6)	.50^c^	.76^c^
Chronic kidney disease or ESKD	9 (3)	12 (4)	.56	3 (12)	2 (6)	.64^c^	.17^c^
Cerebrovascular accident	18 (7)	10 (4)	.10	0	0	NA	.10^c^
Diabetes, type 2	61 (23)	58 (21)	.59	6 (23)	11 (31)	.47	.32
Seizure disorder	4 (2)	11 (4)	.11^c^	0	0	NA	.39^c^
HIV	1 (0)	0	.49^c^	0	0	NA	>.99^c^
Hypertension	85 (32)	75 (27)	.22	8 (31)	10 (29)	.85	.96
Psychiatric problems	3 (1)	5 (2)	.72^c^	0	2 (6)	.50^c^	.27^c^
Substance abuse	8 (3)	8 (3)	.94	0	0	NA	.39^c^
9-1-1 vs non–9-1-1^d^							
9-1-1	245 (93)	252 (92)	.71	26 (100)	34 (97)	>.99^c^	.11^c^
Non–9-1-1	18 (7)	21 (8)		0	1 (3)		
Mobile vs landline call							
Mobile	124 (47)	146 (54)	.13	18 (69)	19 (54)	.24	.13
Landline	139 (53)	126 (46)		8 (31)	16 (46)		
Difficulty identifying the address of the incident	29 (11)	25 (9)	.77	11 (42)	14 (41)	.68	<.001
Location							
Residential (home)	132 (74)	130 (76)	.69	11 (61)	16 (64)	.85	.10
Scene of accident or acute event	47 (26)	42 (24)		7 (39)	9 (36)		
Witness							
Witnessed	87 (33)	93 (34)	.81	11 (42)	7 (20)	.06	.52
Unwitnessed	176 (67)	180 (66)		15 (58)	28 (80)		
Shockability							
Shockable	62 (24)	54 (20)	.29	4 (15)	5 (14)	>.99^c^	.21
Nonshockable	201 (76)	219 (80)		22 (85)	30 (86)		

Abbreviations: ESKD, end-stage kidney disease; IQR, interquartile range; NA, not applicable.

SI conversion factors: To convert pounds to kilograms, multiply by 0.45.

^a^ *P* value of Pearson χ^2^ test for categorical variables and Kruskal-Wallis test for age and weight.

^b^ Others included other with other non-White, non-Hispanic ethnic or cultural backgrounds.

^c^ *P* value of Fisher exact test.

^d^ 9-1-1: call was received as a hand-off from the primary public safety answering point. Non-9-1-1: call arrived directly to the Los Angeles Fire Department call bank through a private number. All calls use the same dispatch system of questions and emergency instructions.

No overall differences in age, sex, or known comorbidities between excluded and included patients or between callers in MPDS and LA-TDS cohorts were observed. No significant differences were found between callers in MPDS and LA-TDS cohorts except that callers with limited English proficiency in LA-TDS cohort had a higher prevalence of heart disease. Furthermore, no significant differences between MPDS and LA-TDS cohorts were found in use of the language line for real-time call translation (MPDS: 7 of 26 [27%] vs LA-TDS: 11 of 35 [31%]; *P* = .80).

As seen in Table 2, for the primary outcome, the prevalence of T-CPR involving callers with limited English proficiency increased from 28% using MPDS to 69% using LA-TDS (odds ratio [OR], 5.66; 95% CI, 1.79-17.85; *P* = .003). For callers with English proficiency, the prevalence of T-CPR increased from 55% using MPDS to 67% using LA-TDS (OR, 1.66; 95% CI, 1.15-2.41; *P* = .007). The interaction of language proficiency as an effect modifier of the improvement in T-CPR using LA-TDS was significant (OR, 1.41; 95% CI, 1.06-3.12; *P* = .03). In the adjusted model, callers with English proficiency were associated with a 69% higher prevalence of T-CPR, whereas callers with limited English proficiency were associated with a 350% greater prevalence of T-CPR.

**Table 2.** Dispatch System Performance on Proposed T-CPR Benchmarks by 9-1-1 Caller English Proficiency

Telecommunicator performance in OHCA	Callers with EP (n = 536)						Callers with LEP (n = 61)					
	MPDS cohort (n = 263)		LA-TDS cohort (n = 273)		OR (95% CI)	*P* value^a^	MPDS cohort (n = 26)		LA-TDS cohort (n = 35)		OR (95% CI)	*P* value^a^
	No.	No. (%)	No.	No. (%)			No.	No. (%)	No.	No. (%)		
Cardiac arrest recognition	256	188 (73)	269	211 (78)	1.32 (0.88-1.97)	.18	26	19 (73)	33	30 (91)	3.68 (0.85-16.02)^b^	.08^b^
Cardiac arrest recognition <1 min	182	35 (19)	207	93 (45)	3.43 (2.16-5.42)	<.001	18	1 (6)	29	9 (31)	7.65 (0.88-66.65)^b^	.07^b^
T-CPR achieved	261	131 (50)	268	160 (60)	1.47 (1.04-2.07)	.03	26	7 (27)	35	22 (63)	4.59 (1.52-13.87)	.007
T-CPR achieved <2 min	131	8 (6)	159	36 (23)	4.50 (2.01-10.07)	<.001	7	0	22	1 (5)	NA	NA

Abbreviations: EP, English proficiency; LA-TDS, Los Angeles Tiered Dispatch System; LEP, limited English proficiency; MPDS, Medical Priority Dispatch System; NA, not applicable; OHCA, out-of-hospital cardiac arrest; OR, odds ratio; T-CPR, telecommunicator-assisted cardiopulmonary resuscitation.

^a^ *P* value of logistic regression.

^b^ *P* value of exact logistic regression.

Performance on proposed telecommunicator benchmarks^18,19^ is shown in Table 2, and elapsed time to key 9-1-1 events (eg, chest compression) is shown in Table 3. Using LA-TDS, among callers with limited English proficiency, there was an earlier description of ineffective breathing. This group had a significant decrease in time to recognition of cardiac arrest (OR, 0.59; 95% CI, 0.41-0.84; *P* = .005) and dispatch of resources (OR, 0.71; 95% CI, 0.54-0.94; *P* = .02) (Table 3). No significant differences between MPDS and LA-TDS cohorts involving callers with limited English proficiency were observed in the rate of sustained return of spontaneous circulation (8 of 26 [31%] vs 11 of 35 [31%]; *P* > .99) or the number of survivors to hospital discharge (3 of 26 [12%] vs 4 of 35 [11%]; *P* > .99).

**Table 3.** Elapsed Time to Key Events in T-CPR for 9-1-1 Callers Reporting Cardiac Arrest

Elapsed time from call receipt to event, s	Callers with EP (n = 536)						Callers with LEP (n = 61)					
	MPDS cohort (n = 263)		LA-TDS cohort (n = 273)		OR (95% CI)^a^	*P* value	MPDS cohort (n = 26)		LA-TDS cohort (n = 35)		OR (95% CI)^a^	*P* value
	No.	Geometric mean (95% CI)	No.	Geometric mean (95% CI)			No.	Geometric mean (95% CI)	No.	Geometric mean (95% CI)		
First caller description of ineffective breathing	200	56.7 (50.6-63.7)	203	52.2 (47.1-57.9)	0.92 (0.79-1.07)	.29	19	100.6 (69.9-144.9)	30	63.3 (45.5-88.1)	0.63 (0.38-1.03)	.07
Median time (IQR)	200	60.0 (56.0)	203	57.0 (58.0)			19	87.0 (110.0)	30	68.5 (73.0)		
9-1-1 Telecommunicator recognition of suspected cardiac arrest	188	106.9 (97.7-116.9)	211	73.0 (66.6-80.0)	0.68 (0.60-0.78)	<.001	19	169.7 (122.1-235.9)	30	99.6 (80.5-123.4)	0.59 (0.41-0.84)	.005
Median time (IQR)	188	103.5 (87.0)	211	70.0 (58.0)			19	175.0 (195.0)	30	101.5 (94.0)		
Dispatch of 9-1-1 resources	245	74.5 (70.4-78.9)	252	54.5 (51.5-57.6)	0.73 (0.67-0.79)	<.001	26	109.8 (90.3-133.5)	34	78.3 (64.2-95.4)	0.71 (0.54-0.94)	.02
Median time (IQR)	245	74.0 (44.0)	252	53.5 (34.5)			26	92.0 (77.0)	34	77.5 (65.0)		
First chest compression in cases in which T-CPR occurred	131	223.9 (210.0-238.8)	160	175.3 (162.8-188.8)	0.78 (0.71-0.87)	<.001	7	278.4 (203.0-381.9)	22	223.8 (188.2-266.3)	0.80 (0.57-1.13)	.20
Median time (IQR)	131	231.0 (94.0)	160	178.5 (126.0)			7	264.0 (174.0)	22	200.0 (134.0)		
First chest compression or EMS arrival or end of call (whichever came first) for all calls	260	229.5 (214.5-245.6)	271	165.4 (153.7-178.0)	0.72 (0.65-0.80)	<.001	26	336.9 (281.0-404.1)	35	254.0 (216.5-298.0)	0.75 (0.59-0.96)	.02
Median time (IQR)	260	240.0 (182.5)	271	164.0 (151.0)			26	345.5 (194.0)	35	266.0 (187.0)		

Abbreviations: EMS, emergency medical services; EP, English proficiency; IQR, interquartile range; LA-TDS, Los Angeles Tiered Dispatch System; LEP, limited English proficiency; MPDS, Medical Priority Dispatch System; OR, odds ratio; T-CPR, telecommunicator-assisted cardiopulmonary resuscitation.

^a^ Ratio of LA-TDS cohort to MPDS cohort.

## Discussion

In this cohort study, the implementation of LA-TDS was associated with a significant and disproportionate increase in T-CPR prevalence involving callers with limited English proficiency. This improvement was achieved using the same 9-1-1 dispatch personnel, minimal retraining, and only a 1-month run-in period (December 2014).

We hypothesized that the disproportionate increase in T-CPR using LA-TDS may be explained by the simplification of the initial portion of the caller interview. This simplification includes decreasing the number of questions, not asking questions that have already been answered, treating vague answers regarding life status (eg, consciousness, breathing normally) as suggestive of agonal breathing, and offering early reassurance that help is on the way. These and other unique features of LA-TDS likely interact in the emergency communication process and are currently under study. No significant change in use of the language line was found between MPDS and LA-TDS cohorts, suggesting that use of interpreter services was not associated with the observed increase in T-CPR. This finding is consistent with a previous study that suggested language lines can promote delays in interaction.^20^ We hypothesize that such delays may have implications for caller-telecommunicator tempo and rapport, caller reassurance, caller confidence, and bystander recruitment to perform more advanced response, such as chest compressions.

A key observation was that callers with limited English proficiency were notably underrepresented in OHCA incidents using both MPDS and LA-TDS. In Los Angeles, half of all residents identify as having Hispanic ethnicity or race/ethnicity other than White, and 19% of residents are considered to have limited English proficiency.^21^ Using MPDS, 9-1-1 callers with limited English proficiency made up only 9.9% of all EMS callers who reported OHCA events, and using LA-TDS, this percentage was 12.8% of all callers reporting OHCA events. This finding suggests a previously unreported gap in knowledge about how, when, and why people with limited English proficiency use 9-1-1. Further studies are needed in communities with a predominance of people with limited English proficiency to examine rates of OHCA reporting, assess attitudes and beliefs about using 9-1-1, and identify barriers to confidently accessing emergency care in these populations.

If T-CPR is as successful as shown in this study in communities with limited English proficiency, additional efforts should be made to integrate targeted teaching on bystander recognition of potential cases of OHCA, early uninterrupted bystander CPR, confident use of 9-1-1, and folowing T-CPR to improve outcomes in culturally marginalized communities. The precise elements of LA-TDS that are associated with increased T-CPR performance in distinct communities also warrant further investigation.

### Limitations

This study has several limitations. First, it took place in a single city with a fire department–based EMS system that is staffed with sworn telecommunicators with training and field experience as emergency medical technicians or paramedics; thus, the setting may not be representative of other 9-1-1 response agencies. Second, Los Angeles has a high percentage of non–English-speaking residents, which may not be representative of other communities. Third, the potential of a Hawthorne effect exists given the political nature and scrutiny of this transition in dispatch systems.^22^ Fourth, the caller party was not an exclusion or assessed in this study, and given the small number of callers with limited English proficiency, it is conceivable that a shift in the distribution of these callers affected the results. Fifth, this study had a small sample size and was underpowered to detect any difference in downstream patient-centered outcomes (eg, survival or functional survival), and we were unable to capture all patient outcomes. Sixth, although the association between language and system was statistically significant, it was not an a priori hypothesis and appeared to be largely attributable to the low T-CPR rates for callers with limited English proficiency using MPDS. The study results are encouraging nonetheless and, even with the small sample size of callers with limited English proficiency, demonstrate T-CPR as an avenue for future study.

## Conclusions

For the given study period, LA-TDS compared with MPDS was associated with increased T-CPR performance in cases in which the 9-1-1 caller had limited English proficiency. Further studies are needed to characterize underrepresented 9-1-1 callers with diverse cultural linguistic backgrounds to improve access, promote activation of the chain of survival, and reduce disparities in cardiac arrest care. The precise elements of LA-TDS that are associated with increased T-CPR performance must also be examined.
